# Risk Factors for Epistaxis in Thoroughbred Flat Races in Japan (2001–2020)

**DOI:** 10.3390/ani13081348

**Published:** 2023-04-14

**Authors:** Fumi Sugiyama, Yuji Takahashi, Motoi Nomura, Yusaku Ebisuda, Kazutaka Mukai, Toshinobu Yoshida

**Affiliations:** 1 Sports Science Division, Equine Research Institute, Japan Racing Association, 1400-4, Shiba, Shimotsuke 329-0412, Tochigi, Japan; 2 Equine Department, Japan Racing Association, 1-1-1, Nishi-Shinbashi, Minato 105-0003, Tokyo, Japan

**Keywords:** exercise-induced pulmonary hemorrhage, epistaxis, Thoroughbred racehorses, risk factor, flat races

## Abstract

**Simple Summary:**

Epistaxis (bleeding from the nostrils) can be caused by exercise-induced pulmonary hemorrhage (EIPH) in Thoroughbred racehorses. EIPH is a common disease in racehorses and 55–75% of horses experience EIPH after racing. In addition, horses with severe EIPH are likely to have poor performance compared to horses without EIPH. The risk factors have been surveyed by several reports, but the results may vary. This retrospective study aimed to identify the risk factors for epistaxis in Japanese flat races between April and September, from 2001 to 2020. Nine factors were significantly associated with epistaxis, including racecourse, body weight, ambient temperature, age, sex, surface condition, racing distance, training center, and race year. While 7 of the factors have been considered in previous studies, we have confirmed that the location of the racecourse and a higher body weight can affect the incidence of epistaxis.

**Abstract:**

We investigated the risk factors for epistaxis in Japanese flat races over a 20-year period. The veterinary records of horses identified as having epistaxis by endoscopy on the race day, and the official racing records of all flat races from April to September between 2001 and 2020, were reviewed. The racecourses (*n* = 10), surface type, surface condition, race class, race distance, race year, sex, age, two training centers, ambient temperature, and body weight on race days were assessed using multivariable logistic regression (*p* < 0.05). Of 475,709 race starts, 616 (1.30 cases per 1000 starts; 95% confidence interval [CI], 1.20–1.40) included an epistaxis event. Nine variables were significantly associated with epistaxis. Seven of the variables have been reported in previous studies: lower ambient temperature, soft surface conditions, shorter racing distances (≤1400 m), increasing age, females and geldings compared to males, training center, and race year. However, two novel variables were identified as significantly associated with epistaxis, increasing body weight per 20 kg (*p* < 0.001, odds ratio [OR], 1.33; 95% CI, 1.25–1.41) and the racecourses that the horses were running at (*p* < 0.001, especially Sapporo [OR; 4.74, 95% CI, 3.07–7.31], Hakodate [OR, 4.66; 95% CI, 3.05–7.11], and Kokura [OR, 4.14; 95% CI, 2.65–6.48] compared to the reference racecourse [Kyoto]). These results can facilitate developing interventions to reduce epistaxis in flat racing.

## 1. Introduction

Epistaxis is a condition defined as bleeding from the nostrils, and it is a common result of exercise-induced pulmonary hemorrhage (EIPH) in racehorses. Previous reports suggest that 55–75% of horses experience EIPH after racing [1,2]. EIPH has been graded from 0 to 4 according to the severity of the bleeding [3], and the most severe form of EIPH is commonly associated with epistaxis. It has been reported that 0.03–0.17% of racehorses show epistaxis after racing [4,5,6] and that the severity of EIPH affects their racing performance [7,8,9]. In many racing jurisdictions, the horses diagnosed with epistaxis are defined as “bleeders” and restrictions are imposed on future participation in racing. For example, the Hong Kong Jockey Club and Racing Australia have banned horses that have recurrent cases of epistaxis [10,11], and, in the Emirates Racing Authority, horses are eliminated from races on their third experience of epistaxis [12]. In the Japan Racing Association (JRA), bleeders are not allowed to participate in a race for 1 month after their first occurrence of epistaxis, 2 months after their second experience, and 3 months for multiple occasions [13]. For horse-racing organizers, a better understanding of the risk factors of epistaxis is important for preventing the occurrence of the disease and improving the health of the horses, both of which reduce the loss of racing opportunities.

The risk factors for epistaxis have been reported previously, including both jump races and flat races in those studies. Horses racing in jump races have a higher odds ratio (OR) compared with those in flat races [4,14], and several reports have revealed that sex and increasing age [5,14,15] are risk factors for developing epistaxis. In addition, the racing distance [14] and the surface conditions in jump races [4,16] have been reported as risk factors of epistaxis, but the results varied among the studies [5]. For EIPH, further analysis has revealed additional risk factors, such as a lower ambient temperature [17,18], accumulated race starts [17,18], and inflammation in the lower airway [19]. Studies have been contradictory in regard to some factors. Longer racing distances have been reported as a risk factor in some studies [18,20], while others have considered that horses racing shorter distances are more likely to develop EIPH [17] or epistaxis [6,14]. The association between epistaxis/EIPH and sex has been reported previously, but the results vary [5,14], with some studies reporting no differences [9,15,17]. This variation in results could be due to differences in sample populations. For example, in Hong Kong, the majority of horses included in races are geldings, whereas racehorses in the UK and Japan are predominantly male and female. Because other differences may exist according to the racing authorities, it is important to investigate the risk factors for EIPH and epistaxis in each racing jurisdiction.

In Japan, the risk factors for epistaxis in 247,564 Thoroughbred horses and 4045 Anglo-Arab horses racing on both flat and steeplechase races between 1992 and 1997 were reported previously [14]. In the study, steeplechase races, age, sex, and race distance were demonstrated as risk factors. However, new factors for EIPH and epistaxis such as ambient temperature [17,18] and surface conditions [4,20] have been reported, but not in Japan. In addition, the number of studies analyzing the risk factors of epistaxis for horses competing in flat races in a large population is limited [4,5]. The aim of this study was to investigate the risk factors for epistaxis in Japanese flat races.

## 2. Materials and Methods

### 2.1. Horses

**Figure 1:**
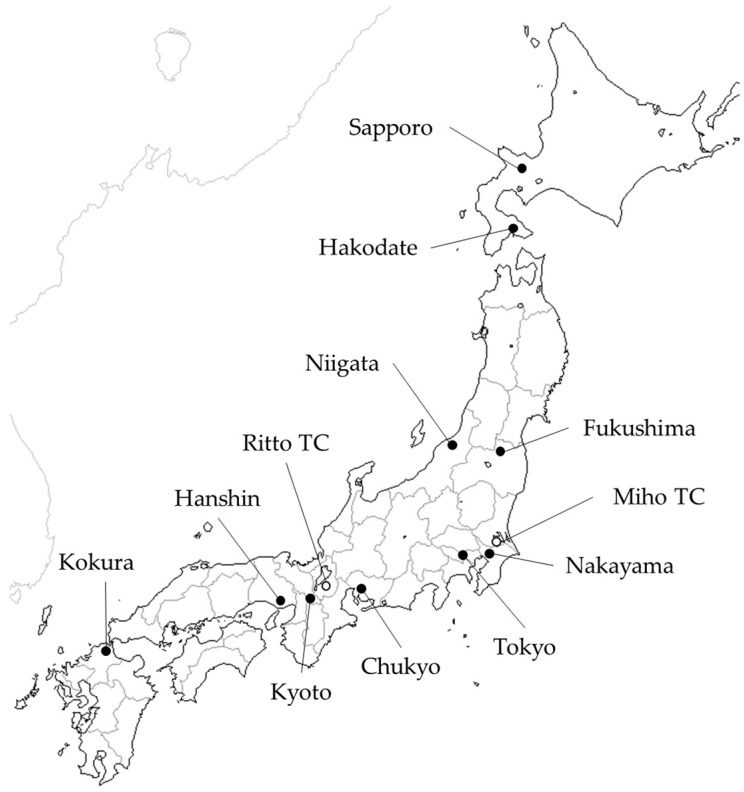
Locations of the 10 racecourses are shown in black circles (●) and 2 training centers (TC) are shown in white circles (○), in the Japan Racing Association. (Created using a map downloaded from Craft MAP [http://www.craftmap.box-i.net] 11 November 2022).

The race information and veterinary records of 941,988 race starts with Thoroughbred horses competing in flat races held by the JRA between 1 January 2001 and 31 December 2020 were collected from the official database. Racing implemented by the JRA was held at 10 racecourses (Sapporo, Hakodate, Fukushima, Niigata, Tokyo, Nakayama, Chukyo, Kyoto, Hanshin, and Kokura) located in a latitude from 34° N to 43° N (Figure 1). Among these 10 racecourses, Sapporo and Hakodate races were held only during the summertime, whereas the other racecourses held races throughout the year. To balance the period of the opening of Sapporo and Hakodate with the other racecourses, the race records from April to September were investigated in this study. All JRA racehorses had to be registered and trained at the Miho (36° N, 140° E) or Ritto (35° N, 136° E) Training Center, according to the outline of the JRA. Therefore, all the horses arrived at each racecourse on the day of the race, or the day before the race. The transportation distance between each training center and the racecourses is shown in Table 1. However, by the judgment of their trainers, some horses were transported to the racecourses several days in advance or stayed at the racecourses between races.

### 2.2. The Definition of Cases and Controls

To identify the risk factors for epistaxis, a retrospective unmatched case-control study was conducted. All horses were led into the racehorse clinic at each racecourse immediately after racing for a veterinary checkup by the JRA official veterinarian. When hemorrhage excretion from the nostril was observed, the horses underwent an endoscopic examination of the trachea. Additionally, by the JRA regulations, stable staff are obligated to inform the official vet when they recognize a hemorrhage from their horse’s nostril. When it was detected at the stable area at the racecourse, the horses were brought to the clinic and went through an endoscopic exam as well. Horses were defined as cases of epistaxis when both conditions, hemorrhage extortion from the nostril and blood excess from the lungs detected by endoscopy, were observed on the race day, while the rest of the horses, which did not have epistaxis, were defined as the controls.

### 2.3. Variables

Ten racecourses were included in this study, with both turf and dirt surfaces. The dirt course was composed of 15 cm of crushed rock as the base layer, 20 cm of mountain sand as the pad layer, and 9 cm of loose sand layer on the surface. The surface condition was defined as “firm” with a firm surface condition, and as “soft” with good, soft, and heavy surface conditions, based on the JRA official race record. The race classes were categorized into 3 groups: “maiden” as a newcomer and maiden, “open” as an open class, and “allowance” for the rest. Race distances were categorized as “≤1400 m”, “1401–1999 m”, and “≥2000 m”. Both the ambient temperature and body weight were recorded at the saddling enclosure approximately 60 min prior to the race by an integral number. The body weight was assessed per 20 kg to clearly define the difference between individuals. Sex was divided into 3 categories, “male” as a sexually intact male, “female”, and “gelding”. The racing age was used as the age variable and categorized into 7 sections: “2”, “3”, “4”, “5”, “6”, “7”, or “≥8” years old, due to the small number of starters over 8 years old. The categories were divided finely to the approximate continuous data. The location of the training stable, “Miho Training Center”, or “Ritto Training Center”, was included in the analysis as well. The race year was categorized by each year to detect the effect of the time course between the race years.

### 2.4. Statistical Analysis

During the study period, 3064 horses from various racing associations other than JRA, such as horses from overseas or from the Japanese municipal racing authorities, were excluded from the statistical analysis.

The ambient temperature and body weight were analyzed as continuous data (per 1 °C and 20 kg, respectively), while the other variables were analyzed as categorical data. Univariable logistic regression analysis was used to obtain the crude OR and associated 95% confidence intervals. For the multivariable analysis, the variables were initially analyzed by a combination of forward- and backward-stepwise procedures and were included in the final model if the *p* value was ≤0.2, as determined by the Score test, and were removed if the value was >0.2, as determined by the Wald Chi-square test.

The two-way interactions between the variables included in the final model were then assessed by backward-stepwise procedure, and the interaction was included in the final model when the *p* value of the Wald test was <0.05. A multivariable logistic regression analysis was performed to assess the risk factors for epistaxis. The variables with a *p* < 0.05 were considered statistically significant and were defined as the risk factors for epistaxis. The fit of the final multivariable model was assessed by the lack of fit test, which compared the log-likelihood for the full model and the current actual fitted model by Chi-square test. The group with the lowest incidence rate was chosen as the reference group to assess the OR in each variable. However, for the race year, 2001 was chosen as a reference for analyzing changes over time. These statistical analyses were performed by JMP (JMP ver. 16.0; SAS Institute, Cary, NC, USA). For the variables in binary categorical form, post hoc power calculation based on this study cohort was conducted on the OpenEpi website (https://www.openepi.com/ 20 August 2022) and the study had a statistical power of 80% to detect an association as significant at the 0.05 level if the OR in the population was ≥1.3.

## 3. Results

The data of 475,709 starts between April and September from 2001 to 2020 were extracted and 616 cases were diagnosed as epistaxis (1.30 cases per 1000 starts; 95% confidence interval, 1.20–1.40). The mean ± SD of the ambient temperature was 23.9 ± 5.9 °C and that of body weight was 468 ± 30 kg. The number of cases and starters in each variable are shown in Table 2. According to the univariable analysis, the racecourse, surface type, surface condition, race class, race distance, race year, sex, age, training center, ambient temperature, and body weight were significantly associated with epistaxis (Table 2).

By a stepwise procedure, the racecourse, surface type, surface condition, race distance, race year, sex, age, training center, ambient temperature, and body weight were chosen to enter the final model. The results of the final multivariable logistic regression analysis for the associations between the variables and the risk for epistaxis are summarized in Table 3. The horses racing at Sapporo, Hakodate, Kokura, Niigata, and Chukyo had a higher risk for epistaxis compared to the Kyoto racecourse. The surface type did not show a significant effect, while the surface condition did: the horses competing on “soft” surface conditions were more likely to develop epistaxis. A short distance (≤1400 m) had a higher OR, compared to middle-length distance (1401–1999 m), whereas no significant difference was found between the middle distance and long distance (≥2000 m). The likelihood *p* value of the race year showed a significant association with epistaxis; however, the Wald *p* value did not show significance when compared to 2001. Females had a much greater risk of epistaxis compared to intact males, while geldings also had a greater risk. An increasing age had a higher risk for epistaxis. The difference in training center had a significant effect on epistaxis. The horses stabled in the Ritto Training Center had a higher OR compared to those at the Miho Training Center. The horses racing in lower temperatures had a higher OR. An increased body weight was associated with an increased OR for epistaxis. No two-way interactions between each explanatory variable in the final model were found. The results of the lack of fit test indicated that the final multivariable model provided a good fit to the data (χ^2^ = 8693.2; 43 degrees of freedom; *p* = 1.00).

## 4. Discussion

The purpose of the present study was to determine the risk factors for epistaxis in Thoroughbred horses competing in JRA flat races. Our results show that 9 factors are significantly associated with epistaxis: racecourse, body weight, ambient temperature, age, sex, surface condition, race distance, training center, and race year.

### 4.1. Racecourse

We showed that the increase in OR was correlated with the racecourse where the horses were running. Among the 5 racecourses that showed a significant association with epistaxis, the OR was remarkably high in 3 racecourses, Sapporo (OR, 4.74), Hakodate (OR, 4.66), and Kokura (OR, 4.14), compared to the reference racecourse. Weideman et al. [5] included the racecourse region as a variable and identified the location of the racecourse as a risk factor. They speculated that the difference in racecourses was caused by altitude, where the racecourses were categorized as sea level, ~1000 m, and ≥2000 m, and concluded that the horses racing at sea level had a greater risk of epistaxis compared to the horses racing at higher altitudes [5]. The altitude of the racecourses managed by the JRA are between 7 m and 64 m, indicating that the difference in altitude is small in Japanese racecourses; therefore, other factors could be responsible for the difference in the OR between racecourses. A previous study has reported temperature, humidity, and air quality as the risk factors [21]. A recent study has analyzed humidity [17,18] and air quality [17] as variables as well; however, they did not find any significance. In our study, the humidity and the air quality at the racecourse were not recorded. However, as indicated from the previous studies, we speculate that the humidity and air quality do not widely affect the differences in racecourses. Transportation has been suspected to affect the horses’ immunological responses and led to many complications, such as gastrointestinal problems, respiratory problems, and death [22,23]. Respiratory disease has been reported as the most common issue related to transportation [24], and some studies have shown strong associations between transportation and an increase in inflammation in the trachea [25,26]. The relationship between EIPH and tracheal inflammation, identified by the tracheal mucus and immunological responses, has been reported in young Thoroughbred horses [19]. Therefore, transportation may have a considerable effect on the respiratory system and may be the cause of epistaxis. Moreover, long-distance transportation, especially longer than 20 h, was reported as an increased risk factor for transport-related health problems [22,24]. According to the JRA survey, the distance between the training centers and the racecourses with relatively high OR were over 1000 km, which suggests that transporting to those racecourses is time consuming, which could have enhanced the chance of epistaxis occurrence in horses racing at the Sapporo, Hakodate, and Kokura racecourses. A longitudinal study is needed for elucidating the association between those parameters and epistaxis.

### 4.2. Body Weight

Based on our results, the OR of epistaxis increased with an increase in body weight. To our knowledge, no survey has included body weight as a risk factor of epistaxis in Thoroughbred horses, which makes this result unique. A recent study on Standardbred horses revealed a correlation between body weight and hemosiderin scores in the bronchoalveolar lavage fluid, indicating heavier horses develop EIPH more frequently than lighter horses [27], which supports our findings. A generally recognized hypothesis of the pathology of EIPH is the failure of the pulmonary capillaries, occurring subsequently to pulmonary capillary hypertension with the correlation of high transmural pressure, often caused by a negative alveolar pressure, during intense exercise [28,29]. These facts suggest that differences in body weight and size might affect the pulmonary arterial pressure, or negative pleural pressure, as a result of a larger respiratory effort. In addition, Lo Feudo et al. [27] considered that the difference in body weight may influence the impact from the fore limb spreading into the thoracic cavity, causing damage to the dorsocaudal area of the lung, which is commonly affected in horses suffering from EIPH [30,31,32]. The theory of impact-induced trauma in the lungs being the cause of EIPH, has been supported by some reports [4,33,34]; however, this theory is not supported by an epidemiological study in Australia [17]. Further study is needed to investigate the relationship between body weight and epistaxis.

### 4.3. Ambient Temperature

Our findings indicate that a decrease in ambient temperature corresponds with the occurrence of epistaxis. This correlation has also been reported by previous studies [17,18] in a relatively small range of temperatures, as well as this study. Crispe et al. [18] considered that exercising in the cold air leads to inflammation in the airway, as reported in human medicine [35], which could increase the risk of EIPH. Others, who have reported the effect of seasons on the incidence of epistaxis and EIPH, concluded winter to be a risk factor for epistaxis and EIPH [5,20], which supports our results. Thus, a reduction in the ambient temperature can be a constant variable that may impact the fundamental aspects of the pathogenesis of epistaxis.

### 4.4. Age

Our results followed most of the previous reports in which increasing age was a risk factor of epistaxis [5,15,19]. Our survey did not include the number of race starts each horse had made, but this does not contradict the hypothesis that accumulating race starts could be a risk factor for EIPH [5,17,18]. The OR peaked at age 6. However, fewer horses older than 6 competed or presented with epistaxis. One possible reason could be that the horses with a history of epistaxis are withdrawn from racing and only healthy horses remain competing. Nonetheless, since the horses over 8 years old still show a significant association with epistaxis, this hypothesis does not fully explain the fluctuation in OR.

### 4.5. Variables Inconsistent with the Previous Literature

In this study, we have revealed a higher OR of epistaxis in females and geldings compared to males. This result aligns with the earlier study from Japan [14], suggesting that the characteristics of Japanese Thoroughbred horses have remained consistent since the former study period. In a previous report, geldings were significantly related to epistaxis [5]; however, some studies did not find a significant relationship between sex and EIPH [15,17,18,21]. This discrepancy may be attributed to the characteristic and the size of the study population in each report.

We have demonstrated that the OR of the “soft” surface condition was higher than the “good” surface condition in this study, which may be supported by the results of a previous study in which the “heavy” surface condition, often due to rain, was considered as a risk factor for the recurrence of EIPH in Brazil [20]. In contrast, Newton et al. [4] did not find a significant association between the surface condition and epistaxis in flat races. However, they noted that this result may be due to a relatively small number of epistaxis occurrences. The power of our dataset was over 80% to detect OR > 1.3, which ensures the validity of the sample size of the current study, verifying that the effect of surface conditions should not be neglected. However, in jump races, a firmer condition was related to an increased odds ratio of epistaxis [16], which could be a contradictory result. The effect of the surface condition on the occurrence of epistaxis may be different between jump races and flat races.

In some previous reports, long-distance races have been concluded as a risk factor for EIPH [18,20]. However, based on our results, the OR of epistaxis increased the most at short-distance races compared with middle-distance races, which have been recognized for epistaxis [14] and EIPH [17] in other studies. An increasing speed has been reported as a risk factor in jump races [16] and barrel-racing horses [25], as well as in flat races [4]. Although steeplechase races are generally slower than flat races, they have been reported to have a higher risk of epistaxis occurrence [4,14]. Furthermore, shorter racing distances have been associated with an increase in speed [36,37] and a higher risk of EIPH or epistaxis [14,17]. These findings may suggest that not only the speed but the acceleration at the start of the race or between hurdles could lead to damage in the lungs. To investigate this hypothesis, a longitudinal survey collecting the individual racing speed or acceleration data by the inertial measurement unit is needed [38].

In this study, we also demonstrated an association between epistaxis and the training center. Previous studies have identified the difference in trainer as a variable. However, those studies did not find any significant association with epistaxis [5] or EIPH [18]. It is possible that the differences in training programs and schedules of training or transportation among training centers may be a possible reason for our results. However, we could not acquire detailed information of those parameters for individual horses.

A significant relationship between the incidence of epistaxis and race year was reported in a previous study [5], but did not suggest any possible reason. In our study, none of the race years were significantly associated with epistaxis compared to 2001. However, race year significantly improved the model fit. Continuous monitoring of the occurrence of epistaxis over time is essential for the entire racing industry.

### 4.6. Limitations

In this study, the horses were detected as having epistaxis by a veterinary checkup or statements from the stable staff. The official veterinarians could only observe the horses once, less than 30 min after the race. After all the horses passed the veterinary checkup, the horses detected as having epistaxis were mainly reported by the stable staff. Although the ideal time to detect EIPH is not established, it is rarely observed within half to 1 hour after finishing the race because of the time taken for the blood to excrete from the nostril. A previous study [17] reported that examining horses too soon after racing was less likely to detect blood. Therefore, we cannot rule out the possibility of overlooked conditions and the number of cases could be underestimated. Nevertheless, due to the low incidence rate of epistaxis, the number of overlooked cases is likely to be small.

In previous studies, the recurrence rate of epistaxis has been reported as 2.06–13% [14,39], which is relatively higher than the incidence rate in all horses. Therefore, it would be better to regulate the effect of recurrent cases. However, since we could not exclude the possibility of horses developing epistaxis during training, we did not consider recurrent cases in this study.

Also, the racing career and racing intervals were not included in this study. We could not track racing careers and intervals in this study, since some horses entered the JRA racing after their career in other Japanese municipal racing authorities. Indeed, some horses stayed at the Sapporo, Hakodate, and Kokura racecourse in between races, and racing intervals might be different from horses going back to their training centers after races.

## 5. Conclusions

On the basis of the results of our current study, the occurrence of epistaxis was associated with the racecourse, difference in body weight, ambient temperature, surface condition, racing distance, age, sex, training center, and race year. Among these factors, ambient temperature, age, sex, surface condition, racing distance, training center, and race year were previously reported. However, the association between racecourse, bodyweight, and epistaxis is a novel finding. The investigation of the risk factors for epistaxis will lead to a further understanding of the pathophysiology of epistaxis and prevention, allowing the horse to achieve a longer and healthier life and prolonging the horse’s racing career.

## Figures and Tables

**Table 1:** The distance (km) between either training centers (TC) and the racecourses.

	Sapporo	Hakodate	Fukushima	Niigata	Tokyo	Nakayama	Chukyo	Kyoto	Hanshin	Kokura
Miho TC	1008	701	265	382	110	77	418	558	598	1118
Ritto TC	1459	1152	718	550	465	480	125	42	82	606

**Table 2:** Numbers of cases and starts implemented by the Japan Racing Association between April and September of 2001 to 2020, with the results of the univariable analysis, stratified by each variable’s category. The Wald *p* value of the reference was not applicable (NA). OR, odds ratio; CI, confidence interval.

Variable	Category	Cases	Starts	Crude OR (95% CI)	Likelihood *p* Value	Wald *p* Value
Racecourse	Sapporo	96	39,895	3.21 (2.11–4.89)	<0.0001	<0.0001
	Hakodate	123	40,825	4.02 (2.67–6.06)		<0.0001
	Fukushima	43	45,646	1.25 (0.78–2.02)		0.3499
	Niigata	77	78,708	1.30 (0.85–2.01)		0.2302
	Tokyo	47	60,495	1.03 (0.65–1.65)		0.8861
	Nakayama	31	38,672	1.07 (0.64–1.78)		0.8016
	Chukyo	23	25,238	1.21 (0.70–2.11)		0.4910
	Kyoto	28	37,293	Reference		NA
	Hanshin	64	64,034	1.33 (0.85–2.08)		0.2065
	Kokura	84	44,903	2.49 (1.63–3.83)		<0.0001
Surface type	Turf	295	256,288	Reference	0.0029	NA
	Dirt	321	219,421	1.27 (1.09–1.49)		0.0029
Surface condition	Firm	425	357,823	Reference	0.0005	NA
	Soft	191	117,886	1.36 (1.15–1.62)		0.0004
Race class	Maiden	237	222,679	0.68 (0.57–0.80)	<0.0001	<0.0001
	Allowance	351	223,106	Reference		NA
	Open	28	29,924	0.59 (0.49–0.87)		0.0081
Distance	≥2000 m	77	74,916	1.08 (0.83–1.41)	<0.0001	0.5638
	1401–1999 m	194	204,000	Reference		NA
	≤1400 m	345	196,793	1.84 (1.55–2.20)		<0.0001
Race year	2001	26	23,216	Reference	0.0335	NA
	2002	35	23,293	1.34 (0.81–2.23)		0.2559
	2003	24	23,951	0.89 (0.51–1.56)		0.6943
	2004	29	23,145	1.12 (0.66–1.90)		0.6775
	2005	27	23,539	1.02 (0.60–1.76)		0.9306
	2006	16	24,459	0.58 (0.31–1.09)		0.0905
	2007	19	23,851	0.71 (0.39–1.29)		0.2588
	2008	28	24,315	1.03 (0.60–1.75)		0.9185
	2009	31	24,486	1.13 (0.67–1.90)		0.6445
	2010	34	24,213	1.25 (0.75–2.09)		0.3894
	2011	41	24,377	1.50 (0.92–2.46)		0.1045
	2012	39	24,280	1.43 (0.87–2.36)		0.1540
	2013	29	24,108	1.07 (0.63–1.82)		0.7911
	2014	26	24,138	0.96 (0.56–1.66)		0.8883
	2015	42	23,993	1.56 (0.96–2.55)		0.0733
	2016	36	23,714	1.36 (0.82–2.25)		0.2369
	2017	31	23,869	1.16 (0.69–1.95)		0.5772
	2018	40	23,463	1.52 (0.93–2.50)		0.0951
	2019	31	22,585	1.23 (0.73–2.07)		0.4440
	2020	32	22,714	1.26 (0.75–2.11)		0.3844
Sex	Male	274	269,281	Reference	<0.0001	NA
	Gelding	27	15,334	1.73 (1.17–2.58)		0.0064
	Female	315	190,694	1.63 (1.38–1.91)		<0.0001
Age	2	26	53,368	Reference	<0.0001	NA
	3	280	239,428	2.40 (1.61–3.59)		<0.0001
	4	153	87,113	3.61 (2.38–5.47)		<0.0001
	5	97	55,607	3.59 (2.33–5.53)		<0.0001
	6	47	26,043	3.71 (2.30–6.00)		<0.0001
	7	8	10,120	1.62 (0.73–3.59)		0.2311
	≥8	5	4,030	2.55 (0.98–6.64)		0.0555
Training center	Miho	264	236,090	Reference	0.0008	NA
	Ritto	352	239,619	1.31 (1.12–1.54)		0.0008
Ambient temperature		Continuous variable per −1 °C		1.03 (1.01–1.04)	<0.0001	<0.0001
Body weight		Continuous variable per 20 kg		1.22 (1.16–1.29)	<0.0001	<0.0001

**Table 3:** The variables chosen by the stepwise procedure, and the result of multivariable logistic regression for epistaxis among horse racings in flat races. OR, odds ratio; CI, confidence interval; NA, not applicable.

Variable	Category	Adjusted OR (95% CI)	Likelihood *p* Value	Wald *p* Value
Racecourse	Sapporo	4.74 (3.07–7.31)	<0.001	<0.001
	Hakodate	4.66 (3.05–7.11)		<0.001
	Fukushima	1.47 (0.89–2.42)		0.1319
	Niigata	1.79 (1.14–2.81)		0.0121
	Tokyo	1.26 (0.77–2.07)		0.3560
	Nakayama	1.31 (0.76–2.25)		0.3291
	Chukyo	1.78 (1.01–3.14)		0.0445
	Kyoto	Reference		NA
	Hanshin	1.32 (0.85–2.07)		0.2186
	Kokura	4.14 (2.65–6.48)		<0.001
Surface type	Turf	Reference	0.1813	NA
	Dirt	1.13 (0.94–1.35)		0.1820
Surface condition	Firm	Reference	0.0170	NA
	Soft	1.25 (1.04–1.50)		0.0158
Distance	≥2000 m	1.23 (0.92–1.63)	<0.001	0.1609
	1401–1999 m	Reference		NA
	≤1400 m	2.04 (1.70–2.44)		<0.001
Race year	2001	Reference	0.0074	NA
	2002	1.22 (0.73–2.03)		0.4438
	2003	0.79 (0.45–1.38)		0.4016
	2004	1.08 (0.63–1.84)		0.7783
	2005	1.00 (0.58–1.71)		0.9965
	2006	0.54 (0.30–1.01)		0.0551
	2007	0.64 (0.36–1.17)		0.1464
	2008	0.93 (0.54–1.58)		0.7776
	2009	1.13 (0.67–1.92)		0.6409
	2010	1.28 (0.76–2.13)		0.3523
	2011	1.40 (0.85–2.30)		0.1855
	2012	1.45 (0.88–2.39)		0.1422
	2013	1.08 (0.63–1.84)		0.7894
	2014	0.96 (0.56–1.66)		0.8842
	2015	1.56 (0.95–2.55)		0.0783
	2016	1.40 (0.84–2.33)		0.1923
	2017	1.18 (0.70–1.99)		0.5462
	2018	1.47 (0.89–2.42)		0.1283
	2019	1.19 (0.70–2.01)		0.5230
	2020	1.20 (0.72–2.03)		0.4837
Sex	Male	Reference	<0.001	NA
	Gelding	1.65 (1.11–2.47)		0.0143
	Female	2.02 (1.69–2.42)		<0.001
Age	2	Reference	<0.001	NA
	3	2.54 (1.68–3.84)		<0.001
	4	3.43 (2.24–5.25)		<0.001
	5	3.46 (2.22–5.39)		<0.001
	6	3.78 (2.31–6.18)		<0.001
	7	1.79 (0.80–4.00)		0.1549
	≥8	2.86 (1.08–7.52)		0.0337
Training center	Miho	Reference	0.0238	NA
	Ritto	1.26 (1.03–1.53)		0.0235
Ambient temperature	Continuous variable per −1 °C	1.04 (1.02–1.06)	<0.001	<0.001

Body weight	Continuous variable per 20 kg	1.33 (1.25–1.41)	<0.001	<0.001


## Data Availability

The data that support the findings of this study are available from the corresponding author upon reasonable request.
